# Effect of antiviral therapy on the survival and incidence of major complications in HBV-associated cirrhotic patients after splenectomy for hypersplenism and portal hypertension

**DOI:** 10.1186/1743-422X-9-273

**Published:** 2012-11-16

**Authors:** Ningqiang Tian, Zhengwen Liu, Mingbo Yang, Zhu Li, Guoyu Zhang, Qunying Han, Na Li, Qianqian Zhu, Yi Lv, Yawen Wang, Fanfan Xing

**Affiliations:** 1 Department of Infectious Diseases, First Affiliated Hospital, School of Medicine, Xi’an Jiaotong University, No. 277 Yanta West Road, Xi’ an, 710061, Shaanxi, China; 2 Department of Hepatobiliary Surgery, First Affiliated Hospital, School of Medicine, Xi’an Jiaotong University, Xi’an, 710061, Shaanxi, China; 3 Institute of Advanced Surgical Technology and Engineering, Xi'an Jiaotong University, Xi’ an, Shaanxi, 710061, China

**Keywords:** Hepatitis B virus, Cirrhosis, Antiviral therapy, Splenectomy, Hypersplenism, Portal hypertension

## Abstract

**Background:**

Splenectomy remains a common approach for the management of hypersplenism and portal hypertension in hepatitis B virus (HBV)-associated cirrhotic patients in China and some other Asian countries. The effects of antiviral therapy on the survival and occurrence of complications in asplenic HBV-associated cirrhotic patients are unknown. This study analyzed the effect of antiviral therapy on survival and occurrence of major complications in HBV-associated cirrhotic patients after splenectomy for hypersplenism and portal hypertension.

**Results:**

Of the 57 eligible patients for analysis, 28 patients received nucleos(t)ide analogs (treatment group) for antiviral treatment after splenectomy, while 29 patients received no antiviral treatment (control group). After a median of 3 years and 9 months, the overall survival and complication-free survival in the treatment group were higher though not statistically significant than those in the control group. Multivariate analysis showed that antiviral treatment was associated with increased but not statistically significant overall survival (hazard ratio (HR): 2.272, 95% confidence interval (CI): 0.952–5.424, *P* = 0.064) and the antiviral treatment was significantly associated with increased complication-free survival of the patients (HR: 7.229, 95% CI: 1.271–41.117, *P* = 0.026). The complication-free survival in patients aged ≤ 40 years was higher than that in patients aged > 40 years in the antiviral treatment patients (*P* = 0.020).

**Conclusions:**

Antiviral therapy initiating after splenectomy may reduce the incidence of complications and tend to improve the survival in asplenic HBV-associated cirrhotic patients, especially in younger patients, supporting the use of antiviral therapy in these patients after splenectomy.

## Background

Hepatitis B virus (HBV) is one of the major causative agents for the development of chronic hepatitis, cirrhosis and hepatocellular carcinoma (HCC) [1]. Antiviral therapy for chronic HBV infection in the immune-active phase and even end-stage cirrhosis and HCC after hepatectomy achieved beneficial effects for the suppression of HBV replication and reduction of disease progression and occurrence of complications [2-8].

Patients with HBV-associated decompensated cirrhosis usually accompany hypersplenism and portal hypertension, which may be attributable to the occurrence of frequent secondary infection and upper gastrointestinal bleeding due to leukopenia and thrombocytopenia and gastroesophageal varices. This may lead to fatal results in some cases. Splenectomy alone or in combination with esophagogastric devascularization is one of the approachs for the treatment of hypersplenism and portal hypertension in cirrhotic patients [9].

Splenectomy is shown to improve the liver function, reverse hypersplenism and reduce portal pressure in cirrhotic patients [9-13]. On the other hand, however, splenectomy may compromise the immune response of cirrhotic patients with chronic HBV infection [14,15]. Therefore, patients after splenectomy represent a subgroup of special individuals in the cirrhotics. Although antiviral treatment with nucleoside or nucleotide (nucleos(t)ide) analogs benefits patients in almost all the disease situation in HBV infection possibly except those in the immune tolerant phase, the effect of antiviral therapy on cirrhotic patients with HBV infection experienced spleen resection is mostly unknown. The aim of this study, therefore, was to explore the effects of antiviral therapy with nucleos(t)ide analogs after splenectomy on the survival and occurrence of major complications in patients with HBV-related cirrhosis.

## Methods

### Patients and data collection

Consecutive patients who were diagnosed HBV-associated cirrhosis and underwent splenectomy for hypersplenism and portal hypertension in the First Affiliated Hospital, School of Medicine, Xi’an Jiaotong University, from January 1, 2006 to December 31, 2010 were considered for inclusion in this retrospective study. Data at splenectomy, including the demographic, laboratory and clinical data, were collected from hospital medical records. The accumulated data of the patients after hospital discharge post splenectomy, including the survival and complications, were then collected by communication with the patients or the family members of the patients using the contact information such as telephone and address and/or by reviewing the case records for those who revisited the doctor. The data were collected as of July 31, 2011.

All the patients were positive for both hepatitis B surface antigen (HBsAg) and antibody to hepatitis B core antigen (anti-HBc). The diagnosis of cirrhosis was established by clinical history of chronic HBV infection with symptoms and signs such as hepatic encephalopathy, abnormal liver function such as hypoalbuminemia, elevated serum total bilirubin, decreased prothrombin time activity, fluctuation of alanine aminotransferase (ALT) and aspartate aminotransferase (AST), and presence of portal hypertension with ascites and esophageal varices, small liver size, irregular liver surface, altered echotexture or CT value and splenomegaly on ultrasonography and/or CT and magnetic resonance imaging (MRI). The diagnosis of cirrhosis was also confirmed by pathological examination of liver tissue biopsy after surgery in some patients. Patients were treated by splenectomy with esophagogastric devascularization according to the severity of esophagogastric varices.

To ensure the comparability, patients aged less than 18 years and diagnosed as HCC before splenectomy were excluded. Patients with infection of other hepatitis viruses and human immune deficiency virus, autoimmune disorders and other diseases unrelated to HBV infection were also excluded.

The study was approved by the Ethics Committee of First Affiliated Hospital, School of Medicine, Xi’an Jiaotong University and performed in accordance with Declaration of Helsinki. Informed consent was obtained from all the patients.

### Categorization of antiviral treatment and control patients

During the study period, antiviral therapy was not routinely recommended for HBV-related cirrhosis patients and the use of antiviral therapy depended on the recommendation of the doctors and the willingness of the patients. This facilitated the collection of data from both patients with and without antiviral treatment. Among all the patients with HBV-related cirrhosis and underwent splenectomy during the study period, we retrieved all the data as described above. Of the patients who had data eligible for analysis, those who commenced their antiviral therapy with nucleos(t)ide analogs within 1 week after splenectomy and the treatment lasted more than 6 months were included as treatment group, and those who did not initiate antiviral therapy after splenectomy throughout the study period were categorized as control group. Patients who received antiviral therapy prior to splenectomy were excluded in terms of antiviral treatment and control patients.

The survival and occurrence of major complications of cirrhosis including ascites, hepatic encephalopathy, variceal hemorrhage, spontaneous bacterial peritonitis, hepatorenal syndrome, development of HCC and deaths after splenectomy during the study period were retrieved and recorded in both the treatment and control groups.

### Statistical analysis

Statistical analyses were carried out using a SPSS16.0 software (SPSS Inc., Chicago, IL, USA). Continuous variables were compared using Student’s *t*-test or Mann–Whitney *U*-test as appropriate. Categorical variables were compared with the *X*^2^ test. Overall survival was defined as the date after splenectomy to the date of death of all causes. Complication-free survival was defined as the date after splenectomy to the date of occurrence of the major complications. Overall survival and complication-free survival were analyzed using the Kaplan–Meier method, and the differences between antiviral therapy-treated patients and the controls were compared using Log-Rank test. Thirteen clinical variables including with or without antiviral treatment were selected for univariate and then multivariate analysis by Cox-regression to identify predictive factors for overall survival and complication-free survival. Statistical significance was set at *P* < 0.05.

## Results

### Background and baseline characteristics

**Figure 1:**
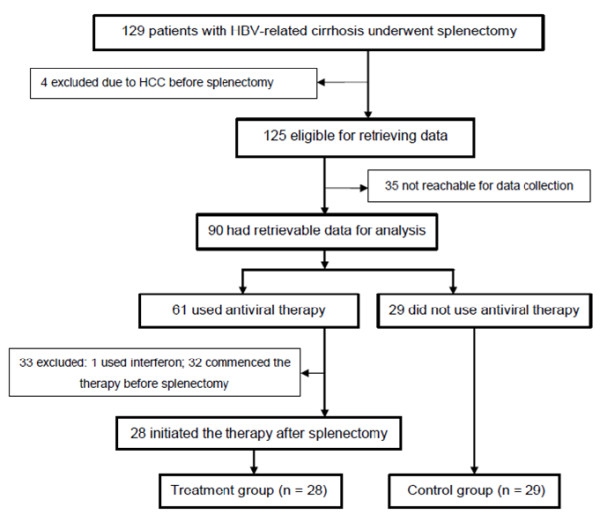
Flow chart of the study population.

From January 1, 2006 to December 31, 2010, a total of 129 patients with HBV-related cirrhosis underwent splenectomy due to hypersplenism and portal hypertension. Of these patients, 4 patients were excluded due to the development of HCC before splenectomy. Of the remaining 125 patients, 35 patients were not reachable to obtain complete data due to the change of contact information. The remaining 90 patients had retrievable data throughout the study period for analysis. Among these 90 patients, 61 patients used antiviral therapy and 29 patients did not. One patient used interferon-α and 32 patients had initiated antiviral treatment before surgery. These 33 patients were excluded in the analysis. Finally, the remaining 28 patients who commenced their antiviral therapy within 1 week after splenectomy and continued the treatment for more than 6 months were included in the antiviral group (treatment group); the 29 patients who had their data collected and did not use any antiviral therapy were included in the control group (Figure 1).

The nucleos(t)ide analogs used in the 28 treatment patients were lamivudine (100 mg/d) in 9, adefovir dipivoxil (10 mg/d) in 11, entecavir (0.5 mg/d) in 4, telbivudine (600 mg/d) in 2, and lamivudine in combination with adefovir dipivoxil in 2 patients. The duration of post-splenectomy antiviral treatment ranged from 7 months to 5 years and 7 months with a median of 3 years and 9 months. Two patients stopped the antiviral therapy (lamivudine) 1 year after the initiation of treatment with no viral breakthrough and occurrence of complication throughout the study period. The remaining patients had used the antiviral treatment throughout the study period. The nucleos(t)ide analogs were safe and all the patients tolerated well with no severe adverse events reported.

The demographics and clinical characteristics of the patients in the treatment and control groups before splenectomy are shown in Table 1. There were no significant differences in the age, distribution of sex, Child-Pugh grade, percentage of HBeAg positivity, HBV DNA load, white blood cell and platelet count, international normalized ratio (INR), ALT and AST levels, serum bilirubin, and albumin level between the 2 groups of patients (Table 1).

**Table 1:** Preoperative demographic and clinical characteristics of the patients in treatment and control groups

	**Treatment group (n = 28)**	**Control group (n = 29)**	***P***
Sex (M:F)	24:4	20:9	0.132
Age [Mean ± SD (range) (years)]	39.75 ± 9.01 (19–54)	42.65 ± 8.56 (23–64)	0.716
Child-Pugh Grade (%)			0.519
A	8 (28.57)	11 (37.93)	
B	13(46.43)	14 (48.28)	
C	7 (25.00)	4 (13.79)	
HBeAg (%)			0.082
Positive	14 (50.00)	8 (27.59)	
Negative	14 (50.00)	21 (72.41)	
HBV DNA (IU/ml) (%)			0.073
<10^3^	4 (14.29)	13 (44.83)	
≥10^3^ -10^5^	10 (35.71)	8 (27.59)	
≥10^5^ - 10^7^	12 (42.86)	6 (20.69)	
≥10^7^	2 (7.14)	2 (6.89)	
White blood cell count (×10^9^*/*L)	2.70 ± 1.85	3.30 ± 4.06	0.854
Platelet count (×10^9^/L)	38.11 ± 14.53	45.80 ± 26.22	0.648
INR	1.12 ± 0.49	1.23 ± 0.21	0.533
ALT( IU/L)	49.00 ± 22.89	50.20 ± 87.24	0.148
AST( IU/L)	47.23 ± 22.60	50.42 ± 77.47	0.079
Serum bilirubin (μmol/L)	28.58 ± 15.76	21.20 ± 7.19	0.139
Albumin (g/L)	32.63 ± 9.16	35.84 ± 6.14	0.129

ALT, alanine aminotransferase; AST, aspartate aminotransferase; HBV, hepatitis B virus; INR, international normalized ratio; IU, international unit; SD, standard deviation.

### Comparison of overall survival between patients with and without antiviral therapy

**Figure 2:**
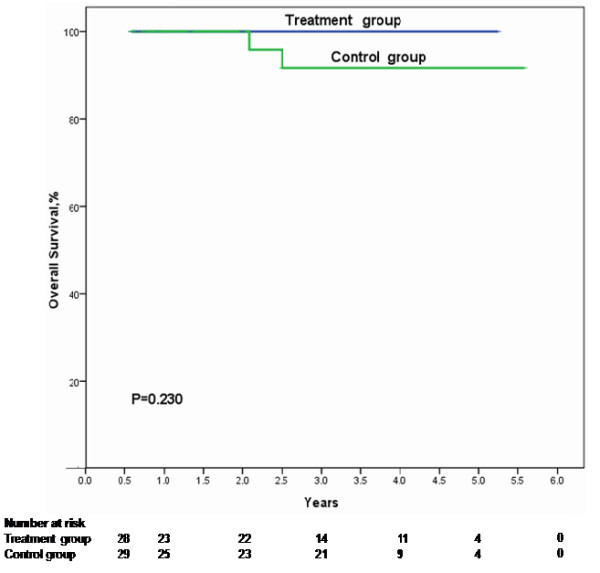
**Cumulative overall survival plotted using the Kaplan–Meier method and compared using log-rank test.** Patients in the treatment group during the study period had higher overall survival than those in the control group but no statistic significance (*P* = 0.230).

No death was observed in the treatment group during the study period. Two patients died in control group. One 54-year-old male patient died of gastrointestinal bleeding 25 months after splenectomy and another 35-year-old female patient died of HCC 30 months after the operation. There was no significant difference of overall survival in patients given antiviral therapy after splenectomy compared with those in the control group. The 1-, 3-, and 5-year overall survival rates in the treatment group were 100.0%, 100.0%, and 100.0%, respectively; in the control group, the 1-, 3-, and 5-year overall survival rates were 100.0%, 90.5%, and 67.9%, respectively (*P* = 0.230, Figure 2).

### Comparison of complication-free survival between patients with and without antiviral therapy

**Figure 3:**
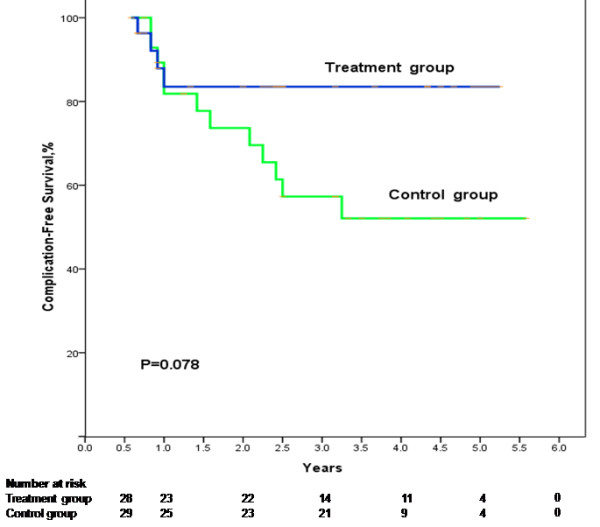
**Cumulative complication-free survival plotted using the Kaplan–Meier method and compared using log-rank test in the treatment group and the control group.** Patients in the treatment group during the study period had higher complication-free survival than those in the control group with no statistic significance (*P* = 0.078).

The complication-free survival rate in the treatment group was higher than that in the control group although the difference was not significant. The 1-, 3-, and 5-year complication-free survival rates in the treatment group were 82.6%, 64.8%, and 64.8%, respectively; in the control group, the 1-, 3-, and 5-year complication-free survival rates were 88.0%, 50.1%, and 25.0%, respectively (*P* = 0.078, Figure 3).

### Factors associated with patient overall survival and complication-free survival

For factors associated with patient overall survival, univariate analysis of the baseline clinical variables revealed no significant factors that could predict overall survival (Table 2). After multivariate analysis, the antiviral therapy was associated with increased overall survival although not statistically significant (hazard ratio (HR): 2.272, 95% confidence interval (95% CI): 0.952–5.424, *P* = 0.064). Serum AST level (HR: 0.273, 95% CI: 0.082–0.907, *P* = 0.034), international normalized ratio (INR) (HR: 0.212, 95% CI: 0.072–0.630, *P* = 0.005) and HBeAg status (HR: 4.823, 95% CI: 1.772–13.129, *P* = 0.002) were factors associated with overall survival (Table 2).

**Table 2:** Univariate and multivariate analysis of the overall survival of patients with HBV-related cirrhosis undergoing splenectomy for hypersplenism and portal hypertension

	**No. of patients**	**Overall Survival (%)**	**3 year**	**5 year**	**Univariate analysis**	**Multivariate analysis**	***P***
		**1 year**			***P***	**HR (95% CI)**	
Antiviral therapy					0.230	2.272(0.952–5.424)	0.064
Yes	28	100.0	100.0	100.0			
No	29	100.0	90.5	67.9			
Gender					0.466	0.359(0.097–1.329)	0.125
Men	44	100.0	95.2	81.4			
Women	13	100.0	88.9	88.9			
Age (years)					0.782	1.513(0.701–3.263)	0.291
≤ 40	26	100.0	94.1	94.1			
> 40	31	100.0	94.4	62.9			
Child–Pugh grade					0.336	1.061(0.482–2.338)	0.883
A	19	100.0	100.0	100.0			
B and C	38	100.0	91.3	76.1			
WBC (×10^9^/L)					0.592	0.352(0.109–1.142)	0.082
≥ 3	20	100.0	90.0	45.0			
< 3	37	100.0	96.0	96.0			
Platelet (×10^9^/L)					0.289	3.951(0.901–17.333)	0.069
≥ 50	13	100.0	85.7	42.9			
< 50	44	100.0	96.4	96.4			
ALT (IU/L)					0.814	0.990(0.289–3.388)	0.987
< 40	28	100.0	94.7	94.7			
≥ 40	29	100.0	93.3	74.6			
AST (IU/L)					0.997	0.273(0.082–0.907)	0.034
< 40	27	100.0	94.1	94.1			
≥ 40	30	100.0	94.4	78.6			
Serum bilirubin (μmol/L)					0.595	1.137(0.262–4.935)	0.864
< 40	49	100.0	93.75	82.0			
≥ 40	8	100.0	100.0	100.0			
Serum albumin (g/L)					0.397	2.603(0.885–7.657)	0.082
≥ 32	41	100.0	92.5	79.3			
< 32	16	100.0	100.0	100.0			
INR					0.716	0.212(0.072–0.630)	0.005
< 1.2	25	100.0	92.8	92.8			
≥ 1.2	32	100.0	95.2	81.6			
HBeAg					0.861	4.823(1.772–13.129)	0.002
Positive	22	100.0	94.1	94.1			
Negative	35	100.0	94.4	75.5			
HBV DNA (IU/ml)					0.964	0.919(0.349–2.421)	0.864
< 10^4^	26	100.0	100.0	75.0			
≥ 10^4^	31	100.0	95.2	95.2			

ALT, alanine aminotransferase; AST, aspartate aminotransferase; HBV, hepatitis B virus; INR, international normalized ratio; IU, international unit; WBC, white blood cell; HR, hazard ratio; 95% CI, 95% confidence interval.

However, for factors associated with patient complication-free survival, univariate analysis of the baseline clinical variables revealed that the antiviral therapy was not significantly associated with complication-free survival (*P* = 0.074, Table 3). The age of the patients at splenectomy was a significant factor (*P* = 0.004, Table 3). However, after multivariate analysis, the use of antiviral therapy was an independent factor associated with complication-free survival (HR: 7.229, 95% CI: 1.271–41.117, *P* = 0.026) and the age (HR: 9.264, 95% CI: 1.806–47.528, *P* = 0.008) remained an independent factor associated with complication-free survival (Table 3).

**Table 3:** Univariate and multivariate analysis of the complication-free survival of patients with HBV-related cirrhosis undergoing splenectomy for hypersplenism and portal hypertension

	**No. of patients**	**Complication-free survival (%)**	**3 year**	**5 year**	**Univariate analysis**	**Multivariate analysis**	***P***
		**1 year**			***P***	**HR (95% CI)**	
Antiviral therapy					0.074	7.229 (1.271–41.117)	0.026
Yes	28	82.6	64.8	64.8			
No	29	88.0	50.1	25.0			
Gender					0.717	1.023 (0.252–4.159)	0.974
Men	44	80.6	61.3	43.6			
Women	13	100.0	55.6	55.6			
Age (years)					0.004	9.264 (1.806–47.528)	0.008
≤ 40	26	95.8	78.8	63.0			
> 40	31	75.0	37.5	37.5			
Child–Pugh grade					0.968	7.848 (0.776–79.315)	0.081
A	19	81.2	54.1	54.1			
B and C	38	87.6	57.1	47.6			
WBC (×10^9^/L)					0.485	5.505 (0.791–38.301)	0.085
≥ 3	20	94.1	65.9	32.9			
< 3	37	80.6	51.6	43.0			
Platelet (×10^9^/L)					0.904	0.307 (0.033–2.834)	0.298
≥ 50	13	100.0	71.4	35.7			
< 50	44	82.1	52.8	44.0			
ALT (IU/L)					0.973	1.959 (0.392–9.794)	0.413
< 40	28	91.7	62.7	62.7			
≥ 40	29	79.1	47.5	28.5			
AST (IU/L)					0.927	0.791 (0.168–3.726)	0.766
< 40	27	86.3	50.8	50.8			
≥ 40	30	84.6	61.1	40.7			
Serum bilirubin (μmol/L)					0.468	1.698 (0.120–24.005)	0.695
< 40	49	85.3	53.3	40.0			
≥ 40	8	85.8	85.8	85.8			
Serum albumin (g/L)					0.688	1.418 (0.276–7.292)	0.676
≥ 32	41	86.11	54.2	38.7			
< 32	16	83.3	62.5	62.5			
INR					0.516	0.372 (0.043–3.228)	0.369
< 1.2	25	100.0	57.1	57.1			
≥ 1.2	32	100.0	71.4	51.0			
HBeAg					0.408	0.573 (0.122–2.696)	0.481
Positive	22	80.0	47.0	31.0			
Negative	35	89.2	64.4	51.5			
HBV DNA (IU/ml)					0.471	1.599 (0.405–6.308)	0.503
< 10^4^	26	81.8	58.4	43.8			
≥ 10^4^	31	88.4	53.7	40.3			

ALT, alanine aminotransferase; AST, aspartate aminotransferase; HBV, hepatitis B virus; INR, international normalized ratio; IU, international unit; WBC, white blood cell; HR, hazard ratio; 95% CI, 95% confidence interval.

**Figure 4:**
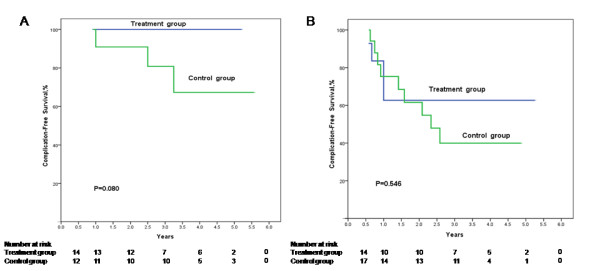
**Cumulative complication-free survival plotted using the Kaplan–Meier method and compared using log-rank test in the patients aged ≤ 40 years (A) and those aged > 40 years (B).** In both the patients aged ≤ 40 years and those aged > 40 years, antiviral treatment was associated with a higher complication-free survival but no statistic significance (*P* = 0.080 and *P* = 0.546, respectively).

**Figure 5:**
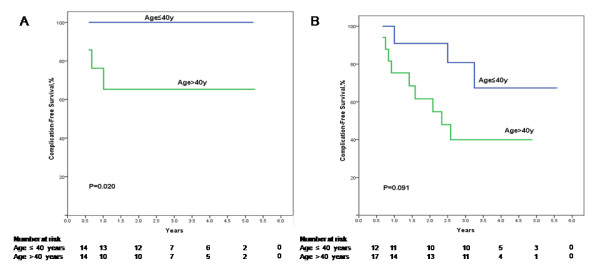
**Cumulative complication-free survival plotted using the Kaplan–Meier method and compared using log-rank test in the treatment patients (A) and control patients (B) according to age.** The complication-free survival in the patients aged ≤ 40 years was significantly higher than those aged > 40 years in the antiviral treatment patients (*P* = 0.020). For the control patients, the complication-free survival in the patients aged ≤ 40 years was also higher than those aged > 40 years but the difference was not significant (*P* = 0.091).

When stratifying patients with and without antiviral treatment according to the age of the patients at splenectomy, complication-free survival rates were improved by antiviral treatment in both patients aged ≤ 40 years (Figure 4A) and those aged > 40 years (Figure 4B). For patients aged ≤ 40 years, the 1-, 3-, and 5-year complication-free survival rates in the treatment group were all 100.0%, and for those of the patients in the control group were 100.0%, 85.71%, and 85.71%, respectively (*P* = 0.080). The 1-, 3-, and 5-year complication-free survival rates for patients aged > 40 years in the treatment group were 71.4%, 40.8%, and 40.8%, respectively, and those of the patients in the control group were 57.1%, 26.0%, and 0%, respectively (*P* = 0.546). When stratifying patients aged ≤ 40 years and those aged > 40 years according to the antiviral treatment or control, the complication-free survival in the patients aged ≤ 40 years was significantly higher than those aged > 40 years in the antiviral treatment patients (*P* = 0.020, Figure 5A). For the control patients, the complication-free survival in the patients aged ≤ 40 years was also higher than those aged > 40 years but the difference was not significant (*P* = 0.091, Figure 5B).

## Discussion

Splenectomy may improve the liver function, reverse hypersplenism and reduce portal pressure and thus decrease the occurrence of complications such as infection, ascites and variceal bleeding in cirrhotic patients [9-13]. However, it may also compromise the immunity of the patients [14,15]. Therefore, best practice care is needed for asplenic patients [16]. For patients with HBV cirrhosis after splenectomy for hypersplenism and portal hypertension, the efficacy and safety of antiviral therapy have not been well documented although the benefit of antiviral therapy with nucleos(t)ide analogs has been demonstrated in most of the disease status of persistent HBV infection [2-8]. In this study, we showed that the overall survival rate and complication-free survival rate in the treatment group was higher than those in the control group although the difference was not statistically significant. This may be the result of the insufficient numbers of patients in our cohort. The univariate analysis showed no significant factors which may predict the overall survival but the age of the patients was associated with complication-free survival. The multivariate analysis showed that the antiviral therapy, though not statistically significant, was associated with increased overall survival and significantly associated to increased complication-free survival of the patients. These results suggest that antiviral therapy also benefits this subgroup of patients and should be implemented in these patients after splenectomy. Of course, it is impartial to commence antiviral treatment before surgery for the HBV cirrhotics. Although antiviral therapy is becoming more and more common in HBV-associated disease, the awareness of antiviral treatment should still be emphasized because it is always not too late to initiate antiviral treatment at any stage of chronic HBV infection.

Serum AST level, international normalized ratio (INR) and HBeAg status were factors associated with overall survival in multivariate analysis. These parameters are reflections of the inflammation and synthetic function of the liver and the characteristics of the virus at splenectomy in the patients and thus may also affect the disease-associated survival after splenectomy. Another independent factor associated to the complication-free survival of the patients was the age of the patients at splenectomy. This is consistent with the long-term observation about the natural history of chronic HBV infection showing that older age at diagnosis is an important determinant of disease progression [17,18]. Notably, our results revealed that, even in the antiviral treatment group, the complication-free survival rate in younger patients was higher than that in older patients, suggesting that younger patients benefit more from the treatment.

There were no serious adverse effects associated with the use of nucleos(t)ide analogs in the present study. Understandably, the most significant concern associated with nucleos(t)ide analogs therapy is the emergence of resistant mutants [19-21], which may lead to a relapse of hepatitis and result in fatal liver failure occasionally. In the present study, we did not determine the emergence of viral resistant mutants. Lamivudine in combination with adefovir dipivoxil was administered in 2 patients *de novo* to prevent the emergence of resistant mutants taking into account of their high HBV DNA levels and the lower rates of resistance and HBV DNA breakthrough as well as the higher rates of ALT normalization with *de novo* combination treatment in chronic hepatitis B patients [22]. None of the patients with nucleos(t)ide analogs developed breakthrough hepatitis in our cohort, probably thanks to the preventive *de novo* combination, the uninterrupted administration of the medicine in the patients and the good compliance in all the patients. With the advent of new and emerging antiviral agents such as tenofovir disoproxil and the optimization and combination of the therapeutics [19,21,23], the emergence of resistant mutants will be well controlled.

One interesting and important issue is the effect of asplenic status on the seroconversion of HBeAg and/or HBsAg with antiviral therapy in the HBV patients. Unfortunately, we were not able to address this issue because of the actual difficulty to retrieve the virological data in most of our patients and to set a comparable group of HBV-related splenic cirrhotic patients. Theoretically, the asplenia status may reduce the seroconversion rate because of the absence of the role of spleen in both innate and adaptive immunity [24-26]. Further studies are necessary to clarify the effect of splenectomy on the seroconversion with antiviral therapy in asplenic patients by comparative observation with splenic patients.

We believe that the antiviral treatment accounts for the improvement in cumulative complication-free survival rate and overall survival rate among patients in the nucleos(t)ide analogs group. However, our study is limited by the small sample size of patients studied, retrospective analysis in design, nonstandardized criteria to use the nucleos(t)ide analogs, lack of addressing the seroconversion and the short observation periods. The statistical power may be insufficient to detect a small susceptible effect of some factors. Therefore, prospective studies in larger sample size of patients with standardized criteria to use the nucleos(t)ide analogs and longer observation periods are needed to clarify the effects of nucleos(t)ide analogs on asplenic HBV cirrhotic patients. In fact, due to the rare condition and the lack of standardized criteria of antiviral therapy for asplenic HBV cirrhosis patients, prospective randomized trials with large numbers of patients seem infeasible, and we believe that our results derived from the retrospective data, do contribute to the understanding of commencement of antiviral therapy in this special subgroup of cirrhotic patients and add novel information for the management of HBV-associated diseases.

In summary, our present study showed for the first time to our knowledge that initiation of antiviral therapy with nucleos(t)ide analogs after splenectomy may reduce the occurrence of major complications and tend to improve the survival in asplenic HBV-associated cirrhotic patients. Multivariate analysis suggests that antiviral treatment and younger age were factors associated with reduced occurrence of complications in the asplenic HBV patients. Younger patients benefit more from the treatment after splenectomy. Our results therefore support the use of antiviral therapy in HBV cirrhotic patients even after splenectomy for hypersplenism and portal hypertension. Larger prospective studies are warranted to confirm our findings and to address the concerns whether antiviral therapy after splenectomy improves survival of the patients and affects seroconversion and viral resistance in the asplenic status.

## Competing interests

The authors declare that they have no competing interests.

## Authors’ contributions

NT involved in conceiving the study and performed data collection and analysis. ZL involved in conceiving and designing the study and writing the manuscript. MY, ZL, and GZ involved in data collection and analysis and revising the manuscript. QH and YL involved in conceiving and designing the study and revising the manuscript. NL, QZ, YW and FX involved in data collection and analysis. All authors read and approved the final manuscript.
